# WormSizer: High-throughput Analysis of Nematode Size and Shape

**DOI:** 10.1371/journal.pone.0057142

**Published:** 2013-02-22

**Authors:** Brad T. Moore, James M. Jordan, L. Ryan Baugh

**Affiliations:** 1 PhD Program in Computational Biology and Bioinformatics, Duke University, Durham, North Carolina, United States of America; 2 Department of Biology, Duke University, Durham, North Carolina, United States of America; 3 Duke Center for Systems Biology, Duke University, Durham, North Carolina, United States of America; The Scripps Research Institute - Scripps Florida, United States of America

## Abstract

The fundamental phenotypes of growth rate, size and morphology are the result of complex interactions between genotype and environment. We developed a high-throughput software application, WormSizer, which computes size and shape of nematodes from brightfield images. Existing methods for estimating volume either coarsely model the nematode as a cylinder or assume the worm shape or opacity is invariant. Our estimate is more robust to changes in morphology or optical density as it only assumes radial symmetry. This open source software is written as a plugin for the well-known image-processing framework Fiji/ImageJ. It may therefore be extended easily. We evaluated the technical performance of this framework, and we used it to analyze growth and shape of several canonical *Caenorhabditis elegans* mutants in a developmental time series. We confirm quantitatively that a Dumpy (Dpy) mutant is short and fat and that a Long (Lon) mutant is long and thin. We show that *daf-2* insulin-like receptor mutants are larger than wild-type upon hatching but grow slow, and WormSizer can distinguish dauer larvae from normal larvae. We also show that a Small (Sma) mutant is actually smaller than wild-type at all stages of larval development. WormSizer works with Uncoordinated (Unc) and Roller (Rol) mutants as well, indicating that it can be used with mutants despite behavioral phenotypes. We used our complete data set to perform a power analysis, giving users a sense of how many images are needed to detect different effect sizes. Our analysis confirms and extends on existing phenotypic characterization of well-characterized mutants, demonstrating the utility and robustness of WormSizer.

## Introduction

Existing methods for measuring nematode size and shape vary in their accessibility, assumptions on nematode morphology, throughput, and interpretability of measured values. As early as 1975, Byerly et al. [1], [2] constructed a machine for rapidly counting and measuring the size of nematodes. Nematodes were suspended in solution and passed between two electrodes. The machine assigned the resistance change caused by a nematode to one of several discrete bins and reported the number of nematodes assigned to each bin. Calibration was required to map each bin to a corresponding value in volume, and the calibration required that all nematodes measured had the same ratio of length to width. The COPAS (Union Biometrica) “worm sorter” is similar to the Byerly machine in that it measures nematodes suspended in liquid. It reports changes to the intensity of a laser beam by passing nematodes. The relative length of each nematode can be estimated by measuring the amount of time the beam is disturbed. The COPAS also returns the optical extinction per nematode: a measure that is sensitive to the opacity, orientation, and size of the animal.

Previous work has measured nematode size from microscopy images. The simplest method manually measured the length and width (at the nematode's center) from images of anesthetized worms and approximated their volume as a cylinder [3]. More recent methods automatically identify and measure nematodes from brightfield images and video. The Worm Toolbox [4] for CellProfiler [5] has been used to measure shape and intensity of various stains in images from high content screens. It can automatically detect and computationally separate tangled worms in these images. Its authors suggest that CellProfiler's existing tools for measuring cellular morphology (such as elliptical width and length) can be applied to nematodes. CellProfiler is open source and written in Python. WormTracker is a motion capture system for tracking a single nematode over time [6]. It produces videos that are subsequently analyzed by its software. It can identify the worm in a given frame, and report its length, width, head and tail width, fatness (ratio of area to length), and several other features. WormTracker is closed source, free for non-commercial use, and written in MATLAB.

We wrote an application, WormSizer, for automated analysis of nematode size and shape. It is open source software written as a plugin for ImageJ/Fiji [7]. WormSizer can therefore be extended and incorporated into existing tools. It is designed to work with a standard stereomicroscope or any other source of brightfield images. WormSizer's target users are biologists, and it has a graphical user interface (GUI). It can detect multiple worms per image and it returns measurements of length, average width, width at the middle of the nematode, and volume in absolute units. Volume estimation is sensitive to the tapering of the worm, unlike the cylinder approximation method. WormSizer also provides a simple and fast interface for quality control of automatically identified nematodes.

We evaluated WormSizer's technical performance in controls, and we used it to analyze growth rate, size and shape in a time course experiment with synchronized populations of various well-characterized mutants and wild-type controls. We show that WormSizer is able to detect relatively subtle differences in size and growth rate, that it can distinguish different morphological phenotypes from wild-type, that it can distinguish dauer larvae, and that it is not confounded by altered behavior of these mutants. In addition to validating WormSizer, we also identified relatively subtle phenotypes not previously described for these well-characterized mutants.

## Results and Discussion

### WormSizer Software

**Figure 1:**
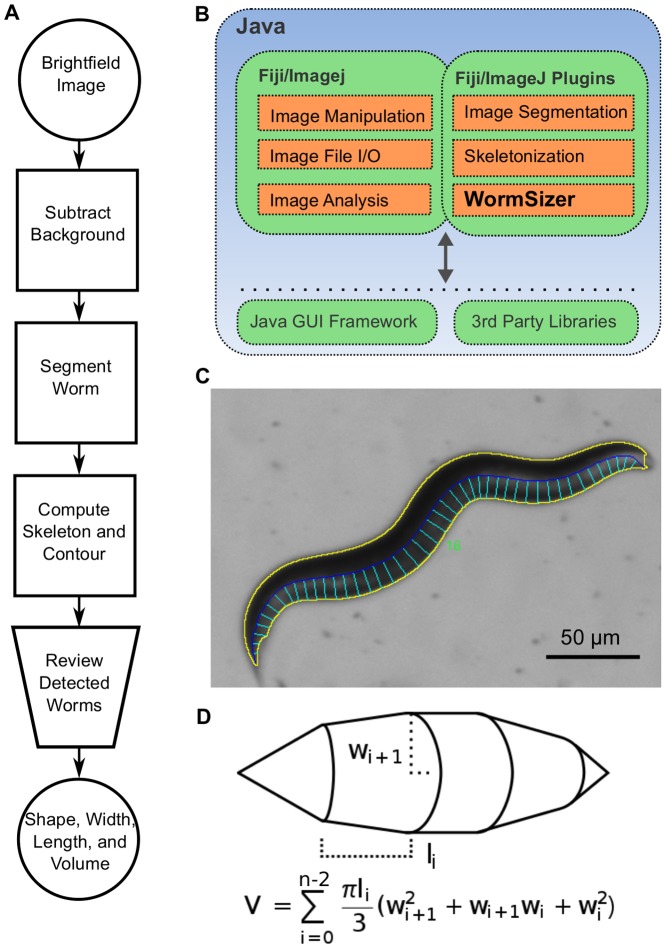
WormSizer: an open source application for detecting nematode size and shape. **A**. The standard workflow of the application. The only manual step (the optional review) is depicted as a trapezoid. **B**. A system diagram depicting how WormSizer interacts with Fiji. WormSizer is written in Java as a Fiji plugin. Fiji plugins written in Java can utilize other Fiji plugins as well as any 3^rd^ party Java library. **C**. The output of WormSizer. Each worm in an input image is identified separately from others. Its contour is highlighted in yellow, its skeleton in blue, and the sampled radii in cyan. This image is shown to users and may be manually passed or failed. **D**. The volume calculation of WormSizer.

WormSizer was written as a plugin for the free, open source, image viewing and processing software Fiji [7], a derivative of the NIH-developed ImageJ [8]. WormSizer can be used with its interactive GUI or as an automated component in another piece of software. It directly links to existing image processing plugins implemented in Fiji. A system schematic is depicted Figure 1.

We designed WormSizer to produce precise quantitative measurements and to require minimal user effort. Prior to imaging, worms are washed off the plates they were grown on and transferred to a clean (i.e. no bacteria) agar plate at low density. This additional step eliminates confounding effects of worm tracks in the image and tangled nematodes. The plate is imaged in brightfield on a stereomicroscope. There is no algorithmic limit on the number of worms per image; each worm will be quantified separately. Tangled or self-intersecting worms will not be correctly quantified; however, WormSizer has an efficient review interface for user screening of the image processing results, enabling removal of these confounding objects.

**Figure 2:**
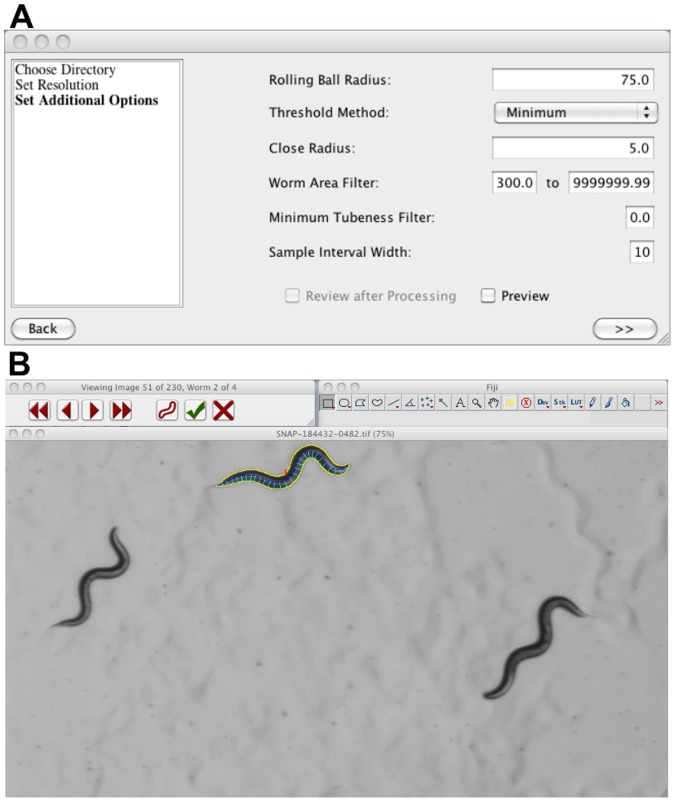
WormSizer screenshots. **A**. The window in which users input the image processing options. Note, there is an option to real-time preview changes in settings. **B**. The review of the image processing results. The user may optionally pass or fail each worm identified in an image. Each worm is shown sequentially (with the option to go backwards or forwards) and may be passed or failed using a keyboard or mouse.

The user interface for WormSizer is straightforward and images are quantified with little setup. The user first chooses a directory containing images (supported file types include byte grayscale TIFF, JPEG, PNG, and BMP images). The user then specifies the absolute scale of the image (microns per pixel) either directly or from previously recorded measurements from a micrometer. Finally, the user configures the parameters for the image-processing algorithm. We found that these parameters rarely need to be changed and were held constant for the experiments reported here, but this flexibility will be valuable with different sources of images. WormSizer automatically detects and measures the nematodes in each image, and its final output are comma-separated (CSV) files containing measurements of volume, length, average width and middle width as well as the settings used in image processing. Optionally, the identified worms in each image can be reviewed and manually annotated pass/fail, providing the user a convenient way to ensure quality control. A view of this interface is shown in Figure 2. This review process is a quick and effective way to remove outliers (e.g. due to worms touching themselves or others) and misidentified objects (e.g. eggs or scratches in the agar).

WormSizer's automatic quantification of nematode size and shape follows a typical image processing workflow: preprocessing, image segmentation, feature extraction and measurement (Figure 1). Preprocessing is used to remove or normalize noise and bias in the initially acquired images in order to simplify image segmentation. Uneven illumination created non-uniform background in our brightfield images. To correct for this, WormSizer uses the rolling ball background subtraction algorithm in Fiji [9]. The algorithm works by treating the image as a 3D surface: the same x-y coordinates of the image are used, but the z coordinate is defined as the intensity of the pixel. Metaphorically, a “ball” of a user-specified radius is rolled all over this surface, and the largest set of continuous points where it touches the surface is considered background. These background points are then set to a constant (average) intensity value. The result is that background regions that are non-uniform but are relatively smooth relative to the ball radius are normalized.

The background correction during the preprocessing step allowed us to use a simple global thresholding approach for segmentation. Global thresholding algorithms work by defining a threshold *t* such that any pixel with intensity below *t* (or above, depending on whether the background is darker or brighter than the object) is considered background, and pixels brighter than *t* are considered part of an object. These algorithms differ in how they compute the threshold *t* from the image data. WormSizer allows the user to specify any of the 14 thresholding algorithms implemented in Fiji's auto threshold [10]. We found that the Minimum algorithm worked well for our images [11]. This algorithm iteratively smooths the intensity histogram of the image until two local maxima corresponding to background and objects are apparent, and then picks a threshold *t* between the peaks such that 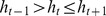 where 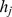 is the number of counts in the smooth histogram for intensity *j*. Continuous pixels with intensity greater than or equal to *t* are identified in the segmented image and are treated as putative worms. These objects may be passed through a filter on area to automatically remove small or large objects.

The skeleton, or medial axis, of each object is computed [12], [13]. For a nematode this should be a single curve; however, due to noise or identifying a non-nematode object, branches may exist. The shortest branches are removed from the original skeleton. The ratio between the length of the pruned skeleton and the original skeleton is used as another user-defined filter on spurious objects. The pruned skeleton is extended on each end along its tangent to the edge of the nematode thereby defining a curve running through the middle of the worm.

**Figure 3:**
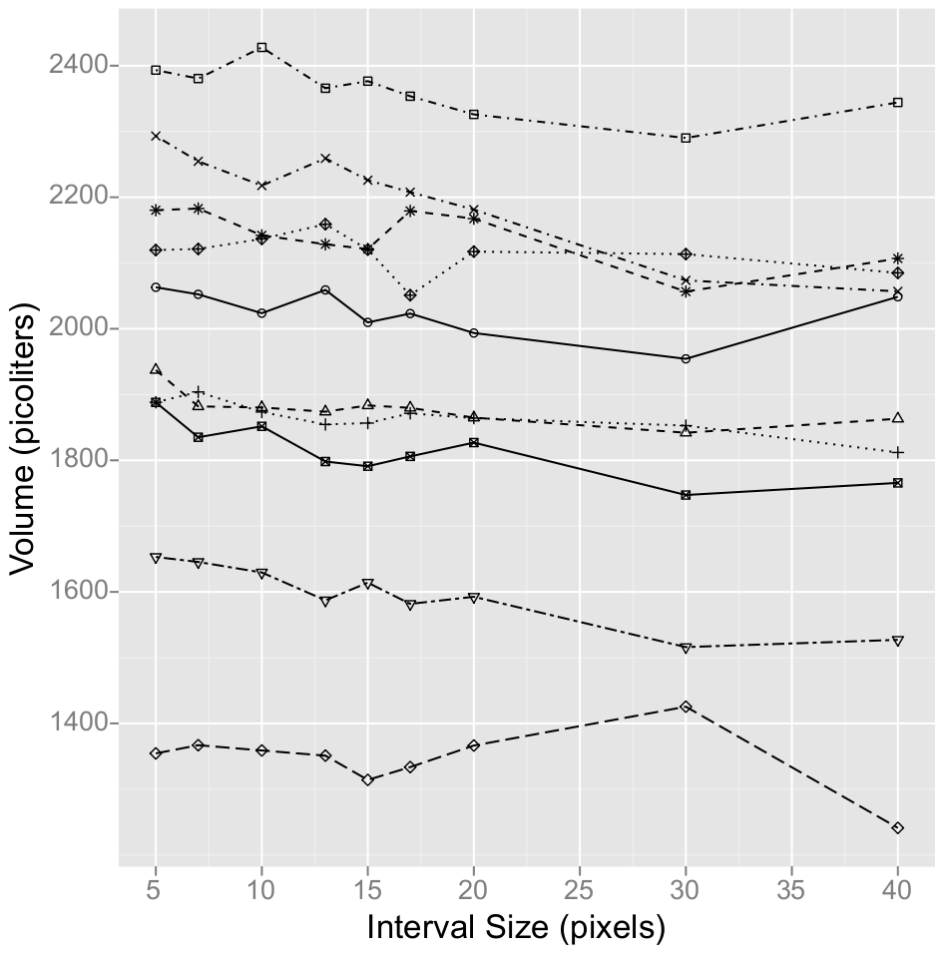
Volume measurement is robust to varying interval sizes. WormSizer was used to segment 10 images of different nematodes (denoted by individual lines in the figure). The interval size (x-axis) was then varied and the resulting calculated volume reported. The average length of the worms was 396 pixels, so an interval size of 40 is approximately 10% of a worm's length. The mean CV of volume across worms across interval sizes was 2.3%.

With the segmented image of the nematode and the skeleton we can measure length, width (middle or average) and volume. Length of the nematode is defined as the length of its skeleton. At a given point along the skeleton, the width of the nematode is calculated as the length of the line between the skeleton and the nematode's edge, orthogonal to the skeleton. If we assume the worm is round (i.e. its cross-section is a circle), then its volume is equal to the 3D integral of its contour rotated around its skeleton. We compute this integral by sampling width at intervals of a fixed size (user-specified) along the skeleton, treating each segment as a frustum of a cone, and summing the volume of these frustums. This is analogous to the trapezoid rule [14] for computing an integral. If the interval size is too small (e.g. one pixel) then error due to the discrete nature of the pixel positions will be introduced, and if it is too large (e.g. half the nematode) the cone segments will be too large to accurately capture the tapering of the worm. We varied the interval size used on individual nematodes and found that the volume calculation is consistent (mean coefficient of variation 2.3% across interval sizes) for reasonable values of the interval size (5 pixels to up to ∼10% of nematode length) (Figure 3). All of the experimental data presented below used an interval size of 10 pixels.

If our assumption of roundness holds, then theoretically our volume measurement should be more accurate and precise than published methods. Cylinder approximation assumes that the volume of the worm is a function of a single measured width (at the middle of the nematode) and the length. This fails to consider how the worm's width tapers along its length. The only assumption our method makes is the radial symmetry of the worm, which we believe is reasonable for worms on an agar surface. However, variation in focus, illumination or worm posture is likely to affect segmentation quality and measurement precision. Since manually focusing the microscope is subjective, we had the same user perform the microscopy for all of our experiments. This issue should be irrelevant on auto-focusing microscopes.

To evaluate WormSizer's technical performance, we quantified segmentation efficiency and measurement precision. For segmentation efficiency, we analyzed a random subset of our experimental images (230 images manually determined to contain 547 worms neither touching themselves or others). The segmentation algorithm automatically identified 80.6% (441) of the worms, and manual annotation of segmentation results as pass/fail ensures specificity. We found the segmentation success rate more than adequate for large-scale studies. In cases where one would want to ensure successful segmentation of a few precious worms (e.g. a time course study of individual worms), we recommend taking multiple images of the same worm across different postures as the worm's illumination will slightly change during movement.

For measurement precision, we evaluated the consistency of WormSizer results by repeated imaging of individual nematodes. We collected data at two time points: 0 hours (arrested L1 stage larvae) and 48 hours (young adults; see below for experimental design). This allowed us to collect measurements at different magnifications and of worms of very different size. For each time point, worms were individually plated and imaged in five independent rounds. Between each round, the microscope's illumination, focus, and zoom were reset and calibrated. Several minutes elapsed between each round, and the nematodes moved between rounds. Nematodes were imaged several times per round in order to guarantee a successfully segmented image (according to user review). Table 1 reports the mean and coefficient of variation (CV) across rounds per nematode. In addition to the WormSizer volume, we report the cylinder-approximation volume using the width at the middle of the skeleton. The mean WormSizer volume and mean CV at 0 hours is 48.1 picoliters (pl) and 11%, and 66.3 pl and 19% for the cylinder approximation method. At 48 hours, the values are 1510 pl and 8.9%, and 2090 pl and 11%. The higher variation at 0 hours reflects the challenge of measuring such small larvae. The heads and tails of these L1 stage larvae tend to be obscured more by the surface of the agar, and this effect is sensitive to focus and posture. Independent of stage, the WormSizer calculation has a smaller CV than cylinder approximation, showing that it is more robust to imaging conditions.

**Table 1:** Repeated measurements of individual worms.

0 hours (L1 arrest)	Mean Volume picoliters (CV)	Mean Cylinder Volume picoliters (CV)	Mean Length µm (CV)	Mean Width µm (CV)
1	48.0 (10%)	65.2 (24%)	201 (2.3%)	20.2 (13%)
2	41.4 (10%)	54.9 (10%)	198 (1.6%)	18.8 (5.8%)
3	47.5 (22%)	65.8 (19%)	201 (4.4%)	20.3 (7.8%)
4	50.2 (13%)	58.5 (10%)	200 (1.9%)	19.3 (4.5%)
5	61.3 (14%)	85.8 (19%)	251 (1.7%)	20.8 (10%)
6	42.7 (13%)	69.0 (18%)	202 (1.7%)	20.8 (9.1%)
7	48.3 (6.9%)	66.4 (25%)	200 (1.8%)	20.4 (11%)
8	40.1 (11%)	54.2 (29%)	191 (2.3%)	18.8 (14%)
9	53.3 (2.6%)	76.4 (15%)	201 (2.4%)	21.9 (8.0%)
Mean	48.1 (11%)	66.3 (19%)	205 (2.2%)	20.2 (9.2%)
Sample CV	15%	21%	6.7%	11%
**48 hours**				
1	1.59E+03 (5.4%)	2.16E+03 (12%)	719 (3.7%)	61.8 (5.9%)
2	1.67E+03 (9.4%)	2.22E+03 (13%)	737 (2.2%)	61.9 (5.6%)
3	1.18E+03 (4.5%)	1.64E+03 (6.5%)	648 (3.1%)	56.7 (2.4%)
4	1.82E+03 (8.8%)	2.48E+03 (10%)	770 (1.6%)	64.0 (4.3%)
5	1.64E+03 (10%)	2.28E+03 (16%)	730 (5.7%)	63.0 (7.3%)
6	1.26E+03 (4.4%)	1.74E+03 (8.0%)	702 (2.5%)	56.1 (4.8%)
7	1.49E+03 (13%)	2.19E+03 (13%)	720 (7.3%)	62.1 (4.1%)
8	1.52E+03 (9.7%)	2.16E+03 (14%)	712 (5.3%)	62.0 (6.6%)
9	1.31E+03 (5.9%)	1.92E+03 (5.0%)	748 (2.5%)	57.1 (2.0%)
10	1.72E+03 (6.7%)	2.33E+03 (11%)	761 (3.5%)	62.4 (4.4%)
11	1.39E+03 (20%)	1.84E+03 (17%)	729 (5.3%)	56.5 (6.8%)
Mean	1.51E+03 (8.9%)	2.09E+03 (11%)	725 (3.9%)	60.3 (4.9%)
Sample CV	21%	23%	8.7%	8.8%

Worms at 0 hours and 48 hours (9 and 11 worms, respectively) were individually plated, imaged, and measured using WormSizer. Worms were measured in rounds (5 rounds total per time point), and the microscope's zoom, focus, and illumination were reset and calibrated according to protocol between rounds. Mean values and CVs are recorded per worm across all five rounds. The volume measurement is as computed by WormSizer; the cylinder volume calculation uses the worm's length and its width at the middle of its skeleton. The reported width is the width at the middle of the worm. The mean values of measurements and CVs are reported across worms. The sample CV is the mean CV across trials from the wild-type control in the main dataset.

The mean CV reported in Table 1 reflects the technical error of WormSizer. The variation observed in a sample population of nematodes will be the sum of technical and biological variation (i.e. the actual physical variation), assuming the two are uncorrelated. Thus, biological variation is the difference between observed sample variation and technical variation. We report the sample population CV as the mean CV across trials of wild-type nematodes at 0 hours (5 trials; Table 2) and another at 48 hours (6 trials; Table 2) (Table 1). Comparing technical and sample variation reveals that both technical error and biological variation contribute significantly to total sample variation.

**Table 2:** The number of samples and biological replicates per strain.

	0 hours		24 hours		48 hours		72 hours	
Strain	Worms	Trials	Worms	Trials	Worms	Trials	Worms	Trials
*daf-2(e1370)*	258	5	698	5	573	5	425	5
*daf-2(e979)*	128	4	497	5	403	5	325	5
*dpy-5(e61)*	200	4	813	4	625	5	338	5
*lon-1(e185)*	104	3	247	4	204	4	96	3
*rol-6(su1006)*	166	4	327	4	283	5	285	5
*sma-9(wk55)*	88	3	69	2	172	4	303	4
*unc-119(ed4)*	60	4	215	5	386	5	363	6
N2 [15°C]	110	5	301	5	306	4	183	4
N2 [20°C]	238	5	809	5	707	6	356	6

Nematodes were discarded after imaging, so each trial is specific to a time point. L1 arrest is denoted as 0 hours, and each subsequent time point is the duration of recovery from arrest with food.

We also compared WormSizer's precision to the COPAS worm sorter. COPAS neither returns a measurement comparable to volume nor is it capable of repeatedly measuring an individual worm. Therefore, we compared the sample CV of WormSizer's length to COPAS' time of flight (TOF) on separate samples of arrested L1 larvae (0 hours). We report a sample CV of 6.7% (Table 1) for WormSizer and a CV of 24% (n = 1091) for COPAS. This result demonstrates that WormSizer is a significantly more precise method for measuring length.

Screenshots of the image processing configuration and user review are shown in Figure 2. The source code repository is publicly available online at: https://github.com/bradtmoore/wormsizer and the compiled software, instructions, and sample data are at: https://github.com/bradtmoore/wormsizer/blob/master/release.zip?raw=true

### Size and Shape Analysis of Synchronized Populations of C. elegans Mutants

With the ability to quantitatively measure size and shape, we decided to investigate several well-characterized mutants with various developmental and behavioral phenotypes. We chose a group of diverse strains that would allow us to evaluate WormSizer's ability to detect differences in size, growth rate and shape. We also included behavioral mutants to determine if it is confounded. These mutants were: *daf-2(e1370)* which increases longevity and stress resistance [15], [16], *daf-2(e979)* which also increases longevity and stress resistance and forms dauers (an alternative larval stage marked by increased stress resistance and a thin morphology) at 20°C [16], *dpy-5(e61)* which has a defect in a procollagen protein that causes the dumpy (short and fat) phenotype [17], *lon-1(e185)* which increases the ploidy of hypodermal cells and causes the long phenotype [18], *sma-9(wk55)* which is part of the TGF-β pathway and has a small phenotype [19], *rol-6(su1006)* which affects a cuticle collagen causing a helical twist and a rolling phenotype [20], and *unc-119(ed4)* which disrupts neuronal function causing paralysis and uncoordinated movement [21].

**Figure 4:**
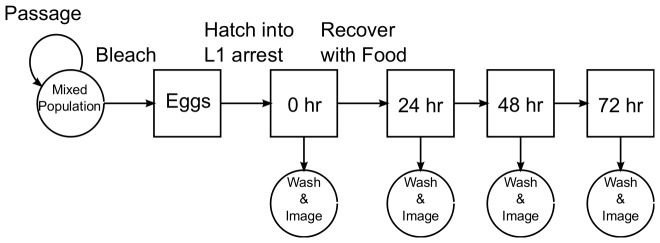
Protocol for imaging staged populations of worms. Each strain is bleached and hatched in the absence of food so it enters L1 arrest (0 hr recovery). Arrested L1 stage larvae are recovered on plates with *E. coli* OP50 as food to initiate post-embryonic development and imaged at subsequent time points. Worms are washed on to clean plates for imaging.

We measured length, middle width and volume during development in synchronized populations (Figure 4). Strains were maintained in standard culture conditions, and gravid adults were bleached to collect eggs. These eggs were cultured for 24 hours in buffer in the absence of food so they hatch and enter L1 arrest. To synchronously initiate post-embryonic development, arrested L1 larvae were recovered on plates with food (*E. coli* strain OP50) at a fixed nematode density. 0 hr recovery corresponds to L1 arrest, and larvae were also sampled at 24, 48, and 72 hours recovery. There are two primary advantages to starting with L1 arrest: arrested larvae are more invariant than any other stage since there is no growth or post-embryonic development during arrest, and recovery from arrest synchronizes larval development. Table 2 contains the number of animals per replicate and replicates per strain. In total, we acquired 9,819 images and measured 11,661 worms (averaging 1.18 worms correctly segmented per image).

**Figure 5:**
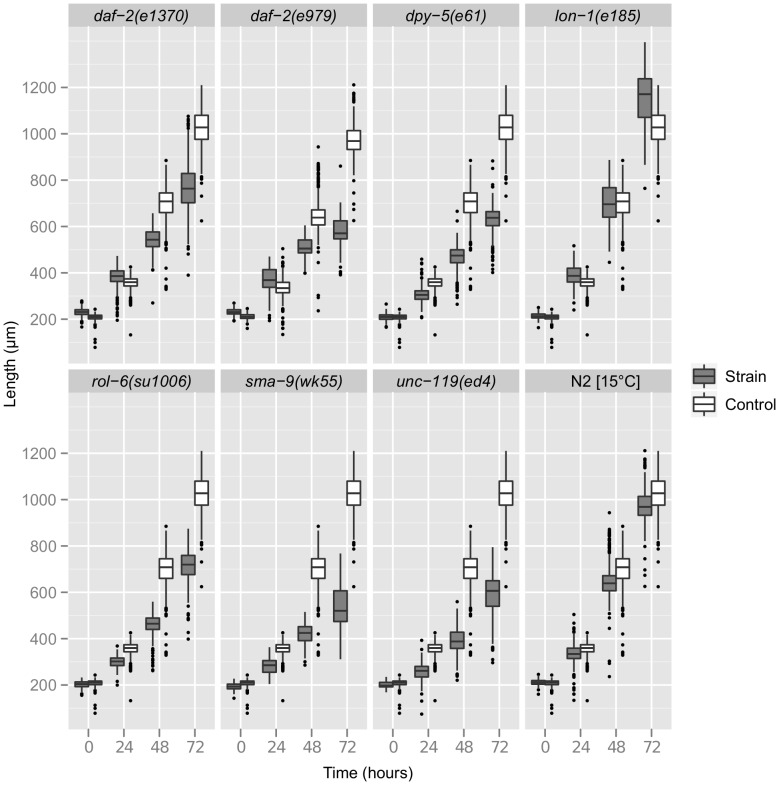
Length measured over time reveals significant differences in growth rate between mutants and wild-type. A boxplot is shown with each strain plotted next to its control over time. The middle hinge of each box is the median, and the lower and upper hinges mark the lower and upper quartiles respectively. The lower whisker is the lower 25% quartile minus 1.5 times the interquartile range (the difference between the upper and lower quartiles). The upper whisker is the upper quartile plus 1.5 times the interquartile range. Outliers that appear outside the whiskers are marked as dots.

Length is the most common size metric reported for *C. elegans*, and WormSizer is capable of robust detection of differences in length. Length is relatively similar among all of the strains at 0 hr recovery (L1 arrest), and differences between each strain and wild-type generally increase over time due to differences in growth rate (Figure 5). *daf-2* mutants grow slower than wild-type as expected [22], especially *daf-2(e979)*, which forms dauers at the assay temperature [16]. Also as expected, *dpy-5(e61)* and *sma-9(wk55)* are shorter and *lon-1(e185)* longer than wild-type [17]–[19]. *rol-6(su1006)* and *unc-119(ed4)* are also both shorter than wild-type, presumably due to compromised movement limiting their ability to feed. We used the Student's *t* test to evaluate statistical significance of observed differences in length (Table 3), and we report the skewness and kurtosis values for volume, which justify the normality assumption.

**Table 3:** Statistical significance of size measurements.

Strain	Time (hours)	Length p Value	Width p Value	Volume p Value	Volume Skewness	Volume Kurtosis
*dpy-5(e61)*	0	1.0	1.0	1.0	0.61	0.89
*dpy-5(e61)*	24	**5.7E-46**	1.0	**7.7E-03**	−0.10	0.20
*dpy-5(e61)*	48	**8.0E-161**	**1.1E-03**	**9.7E-25**	−0.04	0.00
*dpy-5(e61)*	72	**5.0E-84**	1.9E-01	**4.1E-18**	−0.63	0.97
*rol-6(su1006)*	0	1.0	1.0	1.0	0.89	0.49
*rol-6(su1006)*	24	**3.0E-39**	**2.5E-06**	**1.6E-16**	0.02	0.37
*rol-6(su1006)*	48	**9.0E-84**	**5.2E-45**	**1.8E-64**	0.19	0.40
*rol-6(su1006)*	72	**1.1E-73**	**2.3E-26**	**4.6E-53**	0.43	0.19
*lon-1(e185)*	0	1.0	2.8E-01	**1.4E-06**	0.88	0.12
*lon-1(e185)*	24	1.3E-01	**5.1E-08**	1.0	0.51	−0.59
*lon-1(e185)*	48	1.0	**2.5E-37**	**1.4E-21**	−0.17	−0.09
*lon-1(e185)*	72	**7.3E-04**	**4.3E-27**	**3.2E-16**	0.53	1.03
*daf-2(e1370)*	0	**5.4E-19**	**5.0E-06**	**6.1E-17**	0.18	0.79
*daf-2(e1370)*	24	**3.2E-10**	1.0	1.0	0.23	−0.46
*daf-2(e1370)*	48	**1.9E-92**	**2.7E-110**	**9.1E-120**	0.48	1.14
*daf-2(e1370)*	72	**3.7E-42**	**2.0E-84**	**2.0E-87**	0.45	3.52
*daf-2(e979)*	0	**2.0E-11**	1.0	**8.7E-04**	−0.24	−0.46
*daf-2(e979)*	24	9.6E-01	**1.7E-04**	2.3E-01	0.46	1.39
*daf-2(e979)*	48	**7.6E-21**	**5.7E-27**	**8.0E-25**	11.01	164.35
*daf-2(e979)*	72	**1.1E-57**	**6.2E-66**	**4.6E-47**	−0.44	0.09
*sma-9(wk55)*	0	**1.1E-03**	1.0	**5.5E-03**	0.14	0.53
*sma-9(wk55)*	24	**3.4E-30**	**2.0E-03**	**1.2E-12**	−0.09	−0.57
*sma-9(wk55)*	48	**1.7E-71**	**6.4E-29**	**3.6E-43**	−0.10	−0.55
*sma-9(wk55)*	72	**2.3E-103**	**4.1E-77**	**1.9E-91**	0.90	0.69
*unc-119(ed4)*	0	3.5E-01	1.0	5.0E-02	0.00	−0.96
*unc-119(ed4)*	24	**2.2E-30**	**2.8E-05**	**3.5E-21**	0.64	0.89
*unc-119(ed4)*	48	**6.6E-147**	**2.0E-72**	**6.2E-110**	0.62	0.48
*unc-119(ed4)*	72	**1.3E-105**	**7.1E-52**	**1.6E-85**	0.27	−0.28
N2 [15°C]	0	1.0	1.0	1.0	−0.72	1.13
N2 [15°C]	24	1.0	1.0	1.0	0.13	0.04
N2 [15°C]	48	8.4E-01	1.0	1.0	0.70	0.86
N2 [15°C]	72	1.0	**3.8E-09**	**8.5E-07**	0.51	2.68
N2 [20°C]	0				−0.34	−0.15
N2 [20°C]	24				0.46	0.51
N2 [20°C]	48				−0.29	0.13
N2 [20°C]	72				−0.16	−0.40

P values are from a Student's *t* test with the null hypothesis that the mutant (or temperature) is the same as the N2 [20°C] control, except for *daf-2(e979)* where N2 [15°C] is the control, and they are Bonferroni adjusted. Significant (at p<0.01) values are in bold. Values that were adjusted to greater than 1 were set to 1. Sample skewness and excess sample kurtosis are reported for volume. In general, these skewness and kurtosis values justify the normality assumption. *daf-2(e979)* at 48 hours is exceptional, presumably due to the fact that this mutant is undergoing dauer formation at this time, which includes radical change in shape (Figure 7). Nevertheless, the difference in volume for *daf-2(e979)* at 48 hours is qualitatively compelling and significant (*p*<2.2e-16) using Kruskall-Wallis (a non-parametric test).

**Figure 6:**
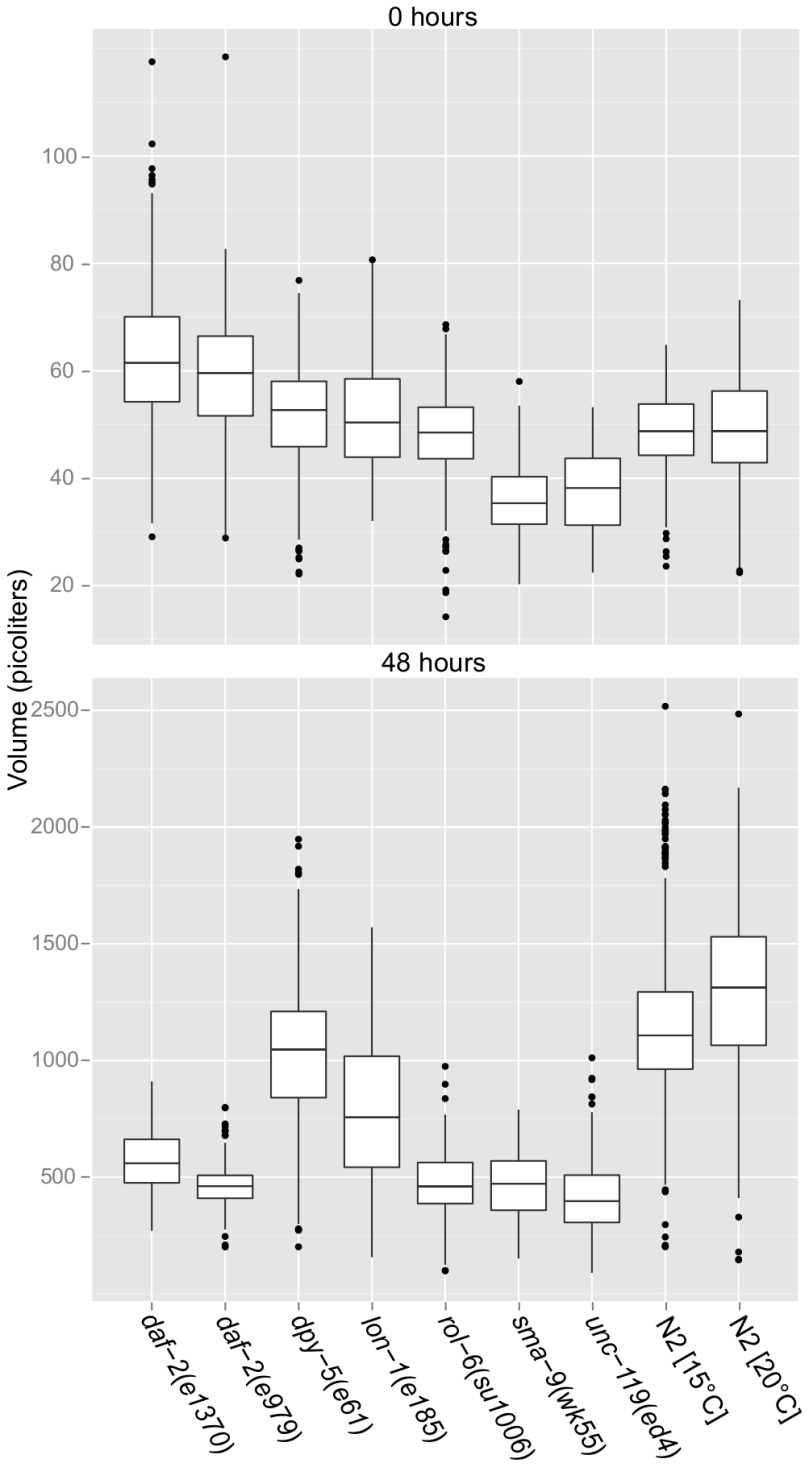
Differences in size upon hatching and after 48 hr growth. A boxplot shows the volume of each strain at 0 and 48 hours. The middle hinge of each box is the median, and the lower and upper hinges mark the lower and upper quartiles respectively. The lower whisker is the lower 25% quartile minus 1.5 times the interquartile range (the difference between the upper and lower quartiles). The upper whisker is the upper quartile plus 1.5 times the interquartile range. Outliers that appear outside the whiskers are marked as dots.

Volume is superior to length for measuring size in that it incorporates width along the animal. Our naïve expectation was that each strain would have a consistent volume during L1 arrest since size at hatching reflects egg size and is independent of growth. However, we found that *sma-9(wk55)* and *unc-119(ed4)* actually have a slightly smaller volume than wild-type during L1 arrest (Figure 6), and these differences are nominally significant (Table 3). We were also surprised to find that both *daf-2* mutants are ∼20% larger upon hatching than wild-type (Figure 6), which is also significant (Table 3). This result has not been reported, and it suggests that insulin-like signaling regulates egg size. Furthermore, these results in L1 arrest with *sma-9(wk55)*, *unc-119(ed4)* and both alleles of *daf-2* show that WormSizer is capable of detecting relatively subtle effects on size, even in such small larvae where precision is compromised (Table 1).

As with length, the mutants tended to be smaller based on volume after 48 hr recovery (Figure 6). *lon-1(e185)* is exceptional in that it is longer (Figure 5) but has smaller volume (Figure 6) due to decreased width (Table 3), highlighting the value of analyzing length, width and volume in conjunction. Likewise, *dpy-5(e61)* is substantially shorter than wild-type at 48 hr recovery but its volume is relatively similar to wild-type despite this (Figure 5, 6 and Table 3).

We also observed an apparent epigenetic effect of temperature on wild-type growth. Because *daf-2(e979)* forms dauer larvae at 20°C, it and a wild-type control had to be cultured at 15°C until recovery from L1 arrest at 20°C. Growth conditions during the experiment were identical for the two wild-type controls, except one had been cultured longer at a cooler temperature until the time series commenced. However, at 48 hr recovery the wild-type nematodes previously grown at a cooler temperature appear to be smaller (Figure 6), and this difference in volume becomes significant by 72 hr (Table 3). This result suggests that nematodes do not alter growth rate immediately in response to temperature increase within their comfort zone, and it demonstrates the ability of WormSizer to detect relatively subtle effects on growth.

**Figure 7:**
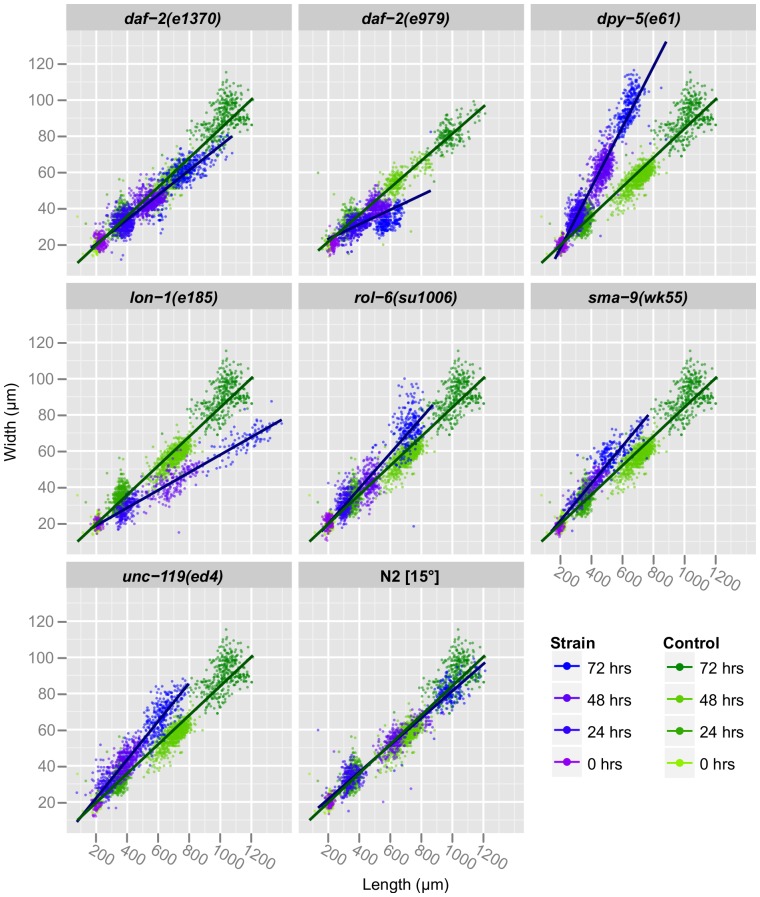
Analyzing length and width over time approximates nematode morphology. Length and middle width of individual animals is plotted for each strain and its control, with time points indicated by color. A linear regression is included for each strain, and the slope of this regression provides an indication of shape.

In addition to the noted differences in growth rate, we observed significant differences in shape between strains. Figure 7 includes scatter plots of each strain's length versus middle width. The slopes of the regression lines show that *dpy-5(e61)* is indeed short and fat and *lon-1* is long and thin. Statistical analysis of regression coefficients indicates that these differences in shape are highly significant (Table 4). We also found that both *daf-2(e1370)* and *unc-119(ed4)* had modest but significant differences in regression slope compared to wild-type, with *daf-2(e1370)* being thinner and *unc-119(ed4)* fatter (Table 4). There is a conserved linear relationship between width and length throughout larval development for each strain, showing that shape is established during embryonic morphogenesis and does not change. *daf-2(e979)* is a notable exception: it actually becomes thinner between 48 and 72 hr as dauer larvae form (Figure 7, Table 3), providing a dramatic illustration of the radial constriction that occurs during dauer formation and suggesting that dauer larvae can be automatically identified by WormSizer.

**Table 4:** Statistical significance of shape measurements.

Strain	Regression Coefficient	p Value
*dpy-5(e61)*	1.7E-01	**0.0E+00**
*rol-6(su1006)*	9.7E-02	2.7E-02
*lon-1(e185)*	4.9E-02	**1.8E-12**
*daf-2(e1370)*	6.8E-02	**4.4E-03**
*daf-2(e979)*	4.1E-02	**3.8E-06**
*sma-9(wk55)*	1.0E-01	2.5E-02
*unc-119(ed4)*	1.1E-01	**6.1E-04**
N2 [15°C]	7.5E-02	4.1E-01
Mixed Stage	8.0E-02	1.0
N2 [20°C]	8.1E-02	

Regression coefficients of length versus width are used to approximate shape. P values are from a Student's *t* test against the weighted difference in regression coefficients with the null hypothesis that the mutant (or treatment) is the same as the N2 [20°C] control, except for *daf-2(e979)* where N2 [15°C] is the control, and they are Bonferroni adjusted. Significant p values (at p<0.01) are in bold.

**Figure 8:**
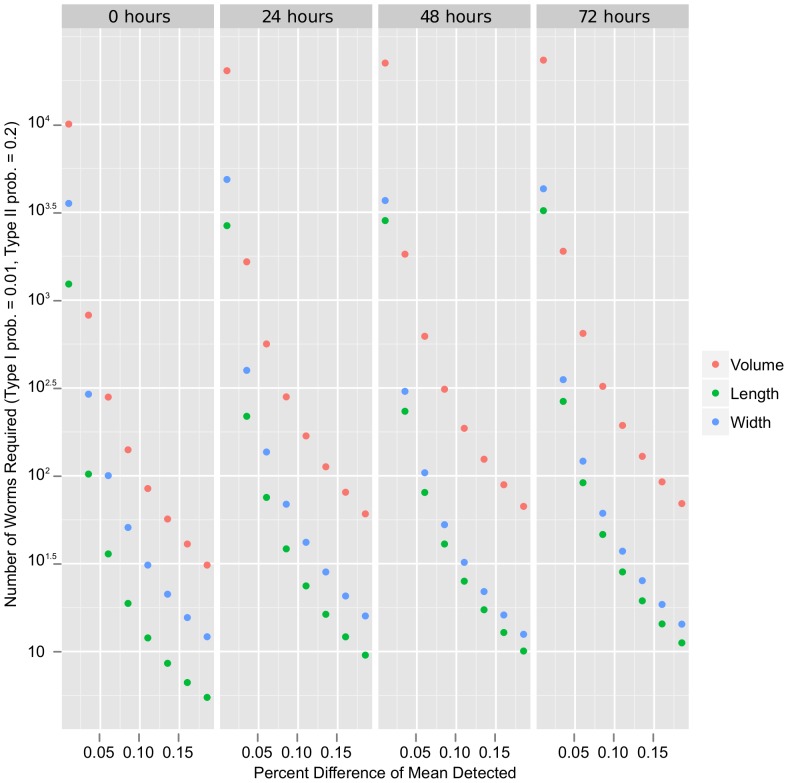
WormSizer can detect differences in size with relatively few samples. Results of a power analysis (t-test, two-sample, two-sided alternative, Type I error probability at 0.01, Type II error probability at 0.2) are plotted for each time point. Less than a hundred individuals are required to detect a 15% difference in volume between populations, and less than three hundred are required for a 10% difference.

One of our goals was to develop a tool capable of producing precise measurements so that statistical power could be achieved with relatively small sample size. Our data encompasses diverse strains with varying phenotypes. By analyzing the variation across all strains in a power analysis, we established a conservative estimate for the number of samples required to detect significant differences between populations. The results are shown in Figure 8. About one hundred worms are required to detect a 15% difference in volume (at a probability of Type II error equal to 0.2), and less than three hundred are required for a 10% difference. Length measurements are more precise than volume, and even fewer samples are required to detect similar differences. The low sample size requirement and the relatively simple imaging and analysis procedure make WormSizer practical and powerful for size and shape assays.

**Figure 9:**
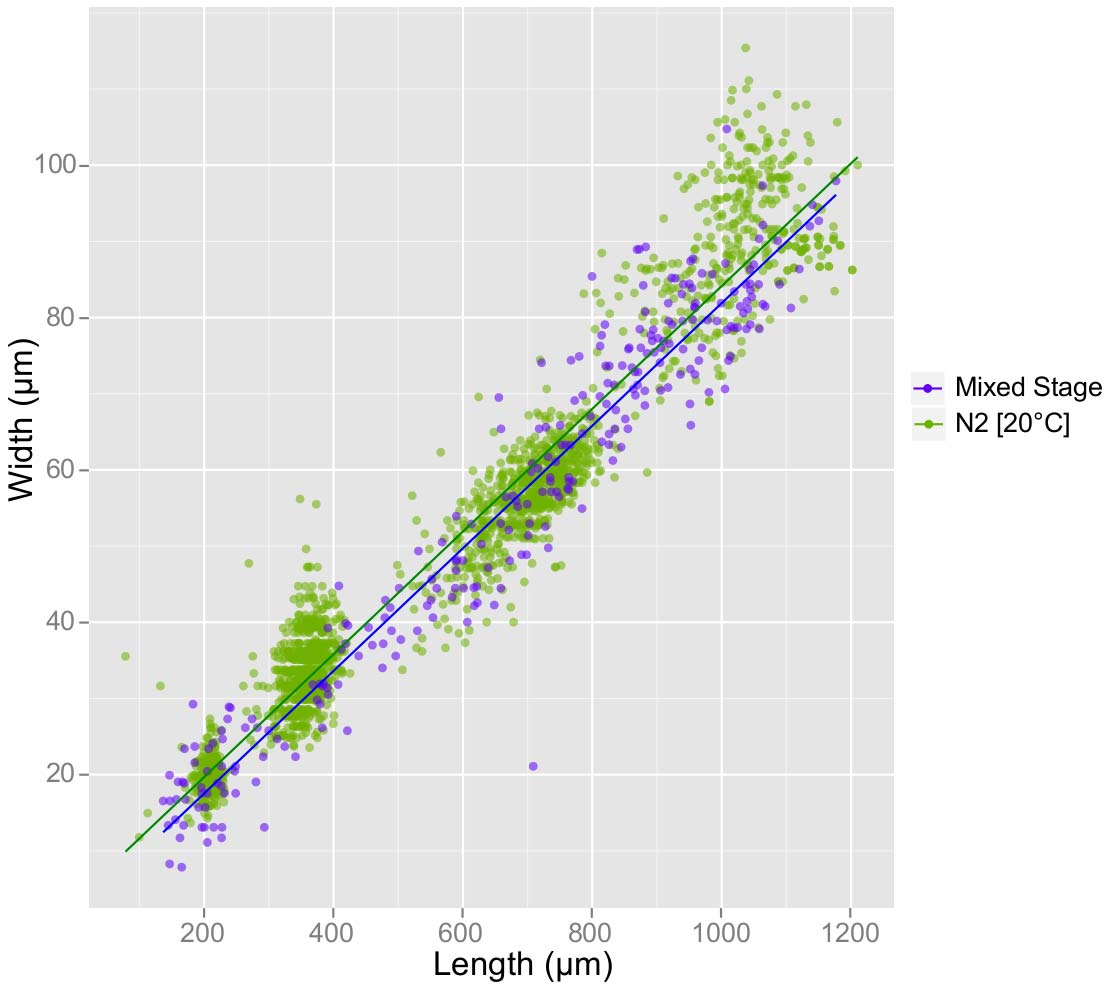
WormSizer can be used on mixed stage populations. Length and width of individual animals are plotted for a mixed staged population (purple) and a staged population over time (green). Shape (length versus width) is not affected by culture method, as revealed by a pair of linear regressions with similar slope (Table 4).

We chose to work with synchronized populations of worms to assess growth rate, but we anticipate the desire to work with images of mixed stage populations. Such an approach does not require staging and could be valuable for high-throughput screens or other applications. We evaluated WormSizer on images of mixed stage cultures to see whether having both young (small) and old (large) worms in a single image would be confounding. Figure 9 shows a length-width scatterplot of a mixed stage population of wild-type animals versus our synchronized wild-type control over time. There is no significant difference between the regression lines of the two populations (Table 4), indicating that WormSizer produces comparable results with synchronized and mixed-stage cultures.

## Conclusions

We have shown the utility of WormSizer for assays of nematode size, growth, and shape. Unlike previous approaches, WormSizer calculates an estimate of volume that is robust to changes in nematode morphology and opacity. The software is easy to use, requires minimal user input, and works with easily obtainable brightfield images. Imaging requires minimal staging (washing nematodes onto a clean plate), and relatively small sample size is needed for sufficient statistical power. An efficient review interface allows any spuriously identified objects (e.g. scratches in the agar, tangled nematodes) to be removed from a user's dataset. We applied WormSizer to a time series experiment with synchronized populations of several mutant strains, and we show it is capable of detecting differences in size, morphology and growth rate. We also show that it is equally effective at analyzing shape in mixed-stage cultures. In addition to reliably quantifying previously characterized phenotypes, we discovered novel phenotypes by virtue of WormSizer's precision: a *sma-9*/SMAD mutant is small upon hatching, *daf*-2/insulin-like receptor mutants are larger upon hatching, and an increase in temperature has a delayed effect on growth rate. WormSizer will be valuable in a variety of contexts, including routine analysis of mutants and growth conditions as well as high-throughput screens.

## Methods

### Strains

Strains: *daf-2(e979)* DR1942, *daf-2(e1370)* CB1370, *dpy-5(e61)* CB61, *lon-1(e185)* CB185, *rol-6(su1006)* HE1006, *sma-9(wk55)* CS1, *unc-119(ed4)* PS3460, and wild-type N2.

### Nematode Husbandry

Worms were cultured at 20°C (except for *daf-2(e979)* and its N2[15°C] control, which were cultured at 15°C) on NGM agar plates with *E. coli* strain OP50. Eggs were collected by bleaching gravid adults [23]. Eggs were suspended in S-basal at a concentration of 1 egg per microliter at 20°C, except for N2[15°C] and *daf-2(e979)* which were at 15°C. These eggs were given 24 hours to hatch and arrest in the L1 larval stage (*daf-2(e979)* and its control were given 48 hours to hatch at 15°C). We designated 0 hr recovery as 24 hr after bleaching (48 hours for *daf-2(e979)* and its control), at which time all strains were recovered on plates with food (OP50) at a density of 1000 nematodes per 10 cm plate. All strains were recovered, including *daf-2(e979)* and the N2[15°C] control, at 20°C.

### Imaging

At 0, 24, 48, and 72 hours recovery, worms were washed onto clean plates (i.e. no *E. coli* food) and imaged on a Zeiss Discovery V20 M2Bio stereomicroscope with digital motorized zoom on an Achromat S 1,0X FWD 63 mm objective (variable zoom). The camera was an AxioCam MRm. The software for acquiring images was AxioVision 4.8.2 (rel 06-2010). The intensity of the illuminator was adjusted so that the mode of the image histogram was centered between 0–255. The exposure time was set to 20 ms. The microscope was manually focused per image. Manual focusing will vary between users; therefore we had a single person perform the microscopy for our experiments. Micrometer measurements at various zooms were manually recorded, and an image scale (microns per pixel) spreadsheet was loaded into WormSizer. The scales for zoom levels not explicitly entered were interpolated from existing values.

### COPAS TOF Data

COPAS produces a spreadsheet reporting data for each event that interrupts the laser sensor. Many of these events may be false positives (e.g. air bubbles). COPAS has a proprietary classification method for eliminating false positives. We filtered the data by only considering events that had an optical extinction (EXT) value greater than a specified cutoff (EXT>20). The CV for TOF using the COPAS filter was 25% (n = 1153) and the CV with our filter was 24% (n = 1091).

### Statistical Test for Difference in Volume, Length, and Width

We report Bonferroni corrected p values for a two-sided *t* test for each measurement against the appropriate N2 control (Table 3). Replicates were subsampled to provide balanced numbers between control and strain, this was performed 1000 times, and the median p value was Bonferroni corrected and reported.

### Skewness and Kurtosis

Sample skewness is defined as,
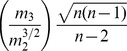
and excess sample kurtosis is defined as,
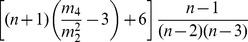
where 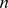 is the sample size and 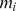 is the *i* ^th^ moment [24].

### Statistical Test for Difference in Regression Slopes

We tested whether the slope of each strain's regression line for length versus width was significantly different from wild-type using a Student's *t* test on the difference between the regression coefficients weighted by the standard error of the difference [25], and report the Bonferroni corrected p values in Table 4.

### Power Analysis

We used the R function *power.t.test* to perform our power analysis. Specifically, the sample size *n* was determined by solving the following for *n*,
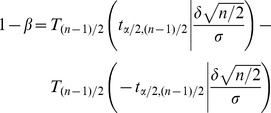
where *n* is the sample size for each of the two groups to be tested, 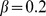 is the bound on Type II error, 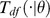 is the cumulative distribution function of the non-central *t*-distribution with *df* degrees of freedom and non-centrality parameter 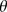, 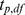 is the point of the central *t*-distribution with *df* degrees of freedom corresponding to an upper-tail probability of *p*, 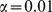 is the bound on Type I error, 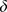 is the absolute difference in sample means which is reported in Figure 8 as a percentage of the wild-type mean, and 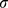 is the standard deviation which is calculated by mean-normalizing each strain and calculating the sample standard deviation across all strains for each time point.
